# BIS-guided sedation prevents the cough reaction of patients under general anaesthesia caused by extubation: a randomized controlled trial

**DOI:** 10.1186/s44158-023-00088-5

**Published:** 2023-02-16

**Authors:** Erfei Zhang, Xiaoying Zhao, Xiaoyan An, Min Wang, Jie Gao, Hailiang Zhang, Ying Li

**Affiliations:** 1 Department of Anesthesiology, The Affiliated Hospital of Yan’an University, Shaanxi Province 716000 Yan’an , China; 2 Department of Anesthesiology, Second Hospital of Shanxi Medical University, Taiyuan, 030001 Shanxi Province China; 3 Department of Operation, The Affiliated Hospital of Yan’an University, Shaanxi Province 716000 Yan’an, China

**Keywords:** BIS-guided sedation, Cough reaction, Peak expiratory flow, Extubation, General anaesthesia

## Abstract

**Background:**

The multiple modes of SARS-CoV-2 transmission including airborne, droplet, contact and faecal–oral transmissions that cause coronavirus disease 2019 (COVID-19) contribute to a public threat to the lives of people worldwide. Heavy aerosol production by coughing and the big peak expiratory flow in patients with respiratory infections (especially SARS-CoV-2) during recovery from general anaesthesia are the highest risk factors for infection in healthcare workers. To perform sedation before extubation significantly reduced the incidence of coughing during recovery from general anaesthesia. However, there are few studies on endotracheal tube removal under BIS-guided sedation in postanaesthesia care unit (PACU). We speculated that the BIS-guided sedation with dexmedetomidine and propofol would better prevent coughing caused by tracheal extubation and reducing peak expiratory flow.

**Methods:**

Patients with general anaesthesia were randomly assigned to Group S (dexmedetomidine was infused in the operating room for 30 min, and the bispectral index (BIS) value was maintained 60–70 by infusion propofol at 0.5~1.5 μg/ml in the PACU until the endotracheal tubes were pulled out) and Group C (no dexmedetomidine and propofol treatment, replaced with the saline treatment). The incidence of coughing, agitation and active extubation, endotracheal tube tolerance and the peak expiratory flow at spontaneous breathing and at extubation were assessed.

**Results:**

A total of 101 patients were randomly assigned to Group S (51 cases) and Group C (50 cases). The incidence of coughing, agitation and active extubation was significantly lower (1(51), 0(51) and 0(51), respectively) in Group S than (11(50), 8(50) and 5(50), respectively) in Group C (*p* < 0.05 or *p* < 0.01, respectively); the scores of cough were significantly reduced (1(1, 1)) in Group S than (1(1, 2)) in Group C (*p* < 0.01); and the endotracheal tube tolerance was significantly improved (0(0, 1)) in Group S than (1(1, 3)) in Group C (*p* < 0.001). The peak expiratory flow at spontaneous breathing and at extubation was significantly reduced (5(5, 7) and 6.5(6, 8), respectively) in Group S than (8(5, 10) and 21(9, 32)) in Group C (*p* < 0.001).

**Conclusions:**

BIS-guided sedation with dexmedetomidine and propofol significantly prevented coughing and reduced peak expiratory flow during recovery from general anaesthesia, which may play an important role in preventing medical staff from contracting COVID-19.

**Trial registration:**

Chinese Clinical Trial Registry: ChiCTR2200058429 (registration date: 09-04-2022) “retrospectively registered”.

## Introduction

The COVID-19 pandemic has been unprecedented for healthcare workers. A study suggested that the respiratory aerosols in exhaled breath are generated by the force of fast airflows in the upper airways that arise when we breathe, talk, cough and sneeze; however, coughing produces more aerosols potentially containing large amounts of COVID-19 [1]. The coughing response caused by tracheal intubation or extubation in patients under general anaesthesia is the most common response in clinical medicine, with an incidence ranging between 38 and 96% [2]. However, a study suggested that coughing during tracheal extubation resulted in 15 times more aerosols than coughing during tracheal intubation [3]. More importantly, the exhaled airflow can travel a distance of approximately 100 cm during tracheal extubation [4], and the amount of aerosol produced is related to peak expiratory flow [5]. Therefore, preventing the cough response and reducing the increase of the respiratory flow during tracheal extubation are the key to reducing the risk of infecting healthcare workers with COVID-19, especially anaesthesiologists, and to preventing secondary COVID-19 infection after endotracheal tube extubation of patients under general anaesthesia with suspected COVID-19. Tracheal tube removal under sedation is performed very early in clinical practice, especially in heart surgery [6]. Postoperative sedation induced with midazolam, and propofol is safe and enables early extubation [7]. Intramuscular dexmedetomidine significantly reduced the incidence of choking reactions from 66 to 20% during recovery from general anaesthesia [8]. Continuous postoperative infusion of remifentanil at 0.3 μg/kg/min reduced the incidence of choking reactions caused by tracheal extubation to 10% in patients under general anaesthesia [9]. Therefore, we speculate that endotracheal tube removal can be performed during recovery from anaesthesia under intravenous sedation. Bispectral index (BIS) monitoring is a standard tool for monitoring sedation levels in the clinic [10]. There are few studies on endotracheal tube removal with BIS-guided sedation in the PACU. The purpose of this study was to explore whether BIS-guided sedation can prevent extubation-induced choking response and increase respiratory flow under recovery anaesthesia.

## Methods

### Trial design

The Affiliated Hospital of Yan’an University, China, organized this RCT. The trial was performed according to the CONSORT-2010 guidelines. The Ethics Committee of the Affiliated Hospital of Yan’an University approved the study protocol (no. 2020042), and all subjects provided written informed consent before the trial.

### Participants and setting

**Fig. 1:**
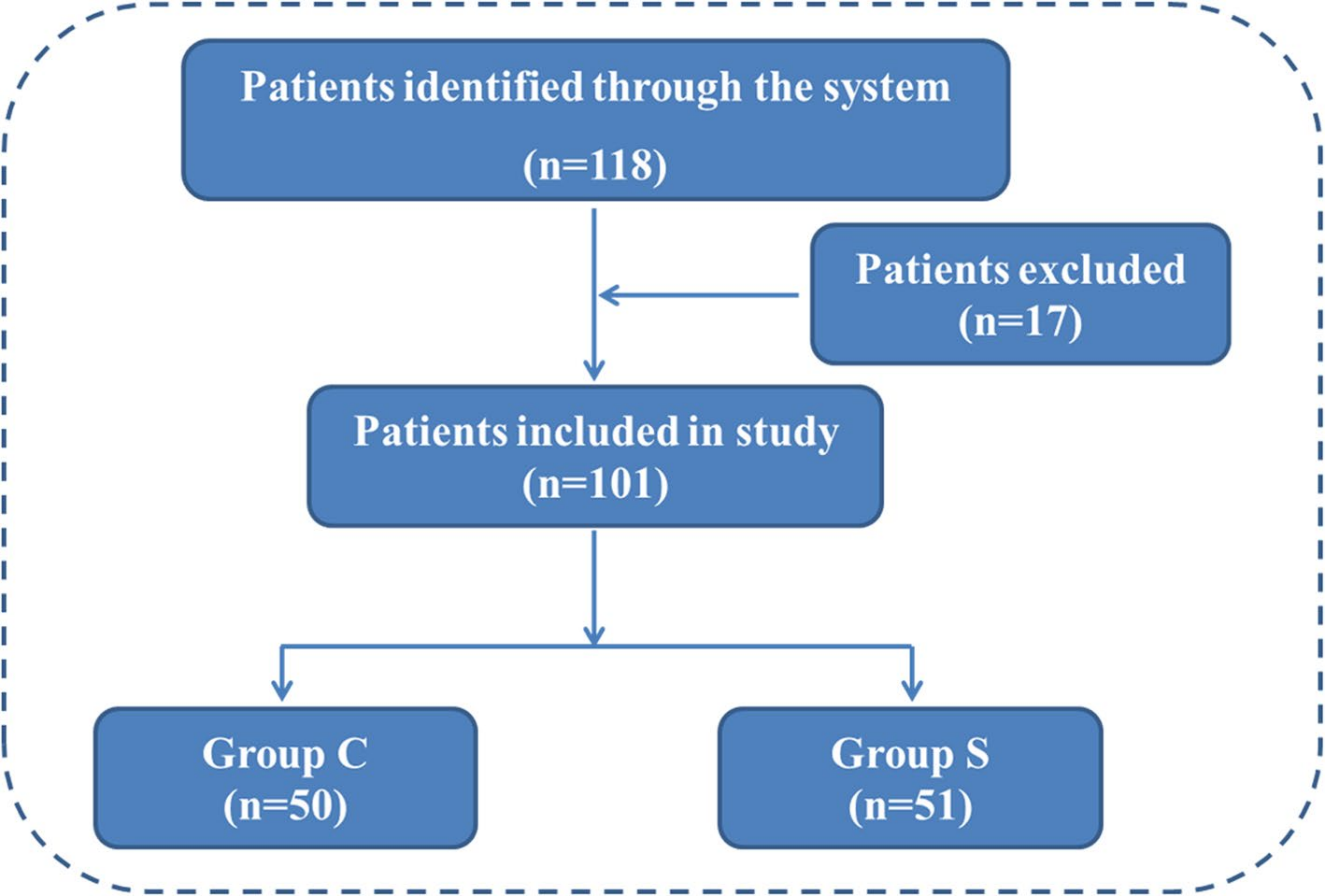
Consolidated standards of reporting trials flow diagram. A total of 118 patients were included in the study according to the inclusion criteria of the trial design. Among them, 17 patients were excluded according to the exclusion criteria. A total of 101 patients were enrolled into the study and randomly divided into 2 groups, 51 patients in Group S and 50 patients in Group C

Patients included in the trial were aged 18~64 years and scheduled for laparoscopic cholecystectomy or cholecystectomy combined with common bile duct exploration under general anaesthesia using endotracheal tube intubation from March 2020 to December 2020. The following major exclusion criteria were used: difficult airway; allergies to lidocaine, tetracaine or any other ingredients in compound lidocaine cream; bradycardia; asthmatic disease; intraoperative bronchospasm; preoperative chronic pharyngitis, cough or other upper respiratory tract lesions; concurrent hypertension with or without drug therapy; bradyarrhythmia; operation time greater than 2.5 h; intraoperative bleeding (> 300 ml); or American Society of Anaesthesiologists (ASA) grade greater than III. We randomly assigned the patients to the sedation group (Group S) and the control group (Group C) at a 1:1 ratio. The primary study endpoint was the incidence of cough caused by endotracheal extubation. The secondary study endpoints were endotracheal tube tolerance assessment during the recovery period, peak airflow velocity at spontaneous respiration recovery and extubation, postoperative cough and sore throat assessment within the first 24 h after extubation. Figure 1 shows a flowchart for the assignment of participants in the study.

### Randomization and blinding

Patient recruitment was performed through our inpatient registration system. Patients who met the inclusion criteria were randomized. Finally, the data analysis excluded patients who met the exclusion criteria. A total of 118 random numbers were generated by IBM SPSS Statistics 25 (IBM Corp. Released 2017. IBM SPSS Statistics for Windows, Version 25.0. IBM Corp, Armonk, NY, USA), and the software randomly divided the 118 numbers into two groups. Cases were enrolled according to the order of enrolment time corresponding to random numbers from small to large, and a random number corresponded to the admission ID number of the patient. A full-time staff member with anaesthesiologist qualification (Investigator A) performed these assignments. When the patients entered the PACU, investigator A controlled the BIS value by drug infusion according to the study design, and propofol infusion was masked by black plastic wrap. Another anaesthesiologist (Investigator B) only performed extubation and collected data until the end of the study. All the collected data were handed over to Investigator A for sorting into different groups, and Investigator C performed statistical analyses and did not know the group information. The patients and Investigators B and C were all blinded to the grouping information.

### Intervention

Based on our experience, we routinely placed all enrolled patients under general anaesthesia. Cisatracurium, remifentanil and propofol were used, and BIS was maintained at 40~60 during surgery. We applied compound lidocaine cream combined with tetracaine as topical anaesthesia to the airway mucosa. Based on a previous study, we applied 2 g of compound lidocaine cream (compound lidocaine cream, 10 g, containing 25 mg each of lidocaine and prilocaine, Tongfang Pharmaceutical Group Co., Ltd. Beijing, China) to the front end of the tracheal tube up to the two black marked lines near the cuff [11] and then sprayed 2 ml of 10% tetracaine injection (tetracaine hydrochloride for injection, 50 mg, Chengdu Zhengkang Pharmaceutical Co., Ltd., Chengdu, China) onto the front end of the tracheal tube at the same location of the compound lidocaine using a small container with a spray function 2 min before endotracheal tube intubation. The endotracheal tube sizes were selected according to our anaesthesiology department protocol (males: *ID*: 7.5~8.0 mm size, females: *ID*: 6.5~7.0 mm size), and the size of the glottis was observed under the video laryngoscope. The recruited patients were treated by anaesthetist A and according to the group information. The patients in Group S were infused with dexmedetomidine at 0.4 μg/kg/h (200 μg of dexmedetomidine was diluted in 50 ml of normal saline, Infusion at 0.1 ml/kg/h speed) for 30 min after entering the operating room. The patients in Group C were infused with normal saline at 0.1 ml/kg/h for 30 min after entering the operating room. After all the enrolled patients entered the PACU, in Group S, the BIS value was maintained at 60~70 indicating that sedation was successfully maintained with an intravenous infusion of propofol (propofol: 0.5~1.5 μg/ml) until the endotracheal tubes were pulled out, as a previous study described [12, 13]; the patients with no sedation in Group C received an intravenous infusion of normal saline (5 ml/h) until the endotracheal tubes were pulled out.

In addition, the depth of anaesthesia was maintained intravenously by continuous infusion of remifentanil and propofol and intermittent intravenous injections of cisatracurium. All continuously infused anaesthetics, including remifentanil, were discontinued when the incision was closed. A multimodal analgesic strategy was employed to achieve postoperative analgesia in this study, in which 40 mg sodium parecoxib was intravenously injected before skin incision, 20 ml of 0.2% ropivacaine was given at an intraperitoneal location, and ~ mL/cm of 0.2% ropivacaine was given at the site of the incision at the end of surgery. No patients required additional analgesics in the PACU. In the study, patients with hypotension and bradycardia were treated with vasopressors and atropine, respectively, depending on the anaesthesiologist’s experience.

### Parameter measurement

The primary study endpoint was the incidence of induced coughing due to endotracheal extubation. The definition of induced coughing was coughing induced by sputum aspiration and extubation in the PACU. The secondary study endpoints were cough scores, incidence of agitation and active extubation, endotracheal tube tolerance assessment score, peak expiratory flow at spontaneous breathing and at extubation, postoperative cough and postoperative pharyngeal pain. Coughing was scored as follows: 0 = no cough; 1 = mild cough; 2 = moderate cough, multiple coughs that lasted shorter than 5 s; and 3 = severe cough, multiple coughs that lasted longer than 5 s. The degree of endotracheal tube tolerance was scored as previously described [14]: 0 = no response during breathing, including spontaneous and mechanical ventilation conditions; 1 = no response during breathing, including spontaneous and mechanical ventilation conditions but slight action response to aspiration of sputum (inconspicuous coughing reaction); 2 = tolerance to mechanical ventilation but moderate action response to aspiration of sputum (single coughing); 3 = tolerance to ventilation, severe coughing reaction (multiple coughs that lasted shorter than 5 s) caused by sputum aspiration; 4 = could not tolerate mechanical ventilation, severe coughing reaction caused by sputum aspiration; and 5 = extubation behaviour. According to previous reports [15], agitation is defined as a patient showing thrashing or violent behaviour or attempts to remove the trachea tube during recovery from anaesthesia. Active extubation was defined as the patient’s attempt to pull the tracheal tube out by hand, without success (special staff ensured that the tube was not removed) during recovery from anaesthesia. The peak expiratory flow was assessed by an electronic peak expiratory flow metre as previously described [16] (Pulmonary Data Services, Louisville, KY, USA). The swivel connector with the suction catheter partially inserted was then attached to the patient’s endotracheal tube, which was in turn connected to a viral/bacterial respiratory filter (GTS, Hong Kong), allowing a pneumotachograph-calibrated Piko-I Electronic Peak expiratory flow metre (Pulmonary Data Services, Louisville, KY, USA to be placed in series as previously described [16]. Postoperative cough was defined as more than 5 spontaneous coughs that lasted longer than 5 s within the first 24 h after extubation, as previously described [17].

### Conditions of endotracheal tube extubation

The following conditions were used for tracheal tube extubation: (1) spontaneous breathing tidal volume greater than 6 mL/kg; (2) respiratory rate ≥ 10 breaths per minute; (3) muscle relaxation monitoring, train-of-four stimulation (TOF) ≥ 0.9; and (4) breathing of air for at least 10 min with SPO_2_ not lower than 95%.

### Sample size calculation

According to our preliminary study that was not published, the incidence of cough response induced by extubation was 25% in Group C and 2% in experimental Group S. We set *α* = 0.05, and the test power was 0.85, with a sample drop-out rate of 20%. Using PASS 15, we calculated a minimum sample size of 59 cases in each group (a total of 118 cases).

### Statistical analysis

Discrete variables are expressed as frequencies (%) and were analysed by the chi-square test or Fisher’s exact test. The Shapiro–Wilk test was used to test the normality of the continuous variables followed by Student’s *t*-test for normally distributed data, and the Wilcoxon rank-sum test was used for comparison of the data that were not normally distributed. A *p*-value < 0.05 was considered to be statistically significant. SPSS 25 was used to process the data.

## Results

### Patients

A total of 118 patients at the Affiliated Hospital of Yan’an University were enrolled in the randomized trial. Seventeen patients were excluded from this study, including 8 patients in Group S (difficult airway: 2 patients; preoperative chronic pharyngitis: 2 patients; allergies to compound lidocaine cream: 1 patient; patients with concurrent hypertension: 2 patients; operation time longer than 2.5 h: 1 patient) and 9 patients in Group C (difficult airway: 3 patients; preoperative chronic pharyngitis: 1 patient; allergies to compound lidocaine cream: 1 patient; patient with concurrent hypertension: 2 patients; operation time longer than 2.5 h: 2 patients). The study started on March 1, 2020, and ended on December 31, 2020. The endpoint of the study was the incidence of cough within the first 24 h after extubation. There were no significant differences in the baseline characteristics (such as age, weight, BMI, sex, smoking, operation time and anaesthesia time) of the patients between the groups (Table 1).

**Table 1:** Patient characteristics

	Group S	Group C	*t*	*p*
Age (year)	43 ± 10	43 ± 8	0.1157	0.454
Weight (kg)	70 ± 8	68 ± 11	0.7139	0.4771
BMI	26 ± 3	25 ± 3	1.120	0.1327
Sex (male *n* (N))	15 (51)	14 (50)	0.080	0.777
Smoking (*n* (N))	10 (51)	12 (50)	0.286	0.593
Operation time (min)	53 ± 23	53 ± 25	0.1629	0.4355
Anaesthesia time (min)	68 ± 23	67 ± 24	0.1953	0.6642

### Primary outcome

We found that sedation significantly reduced the incidence of induced coughing, and the incidence of induced coughing 1 (51) in Group S was lower than that in Group C 11 (50), *p* = 0.006 (Table 2).

**Table 2:** Findings during recovery from general anaesthesia and recovery outcomes

	Group S	Group C	*χ*^2^/k/z/t	*p*
Incidence of induced cough#	1 (51)	11 (50)	7.530	0.006
Scores of cough^b^	1 (1, 1)	1 (1, 2)	−2.782	0.005
Tracheal tube tolerance^b^	0 (0, 1)	1 (1, 3)	−4.456	< 0.001
Incidence of emergence agitation*	0 (51)	8 (50)	-	0.027
Active extubation rate*	0 (51)	5 (50)	-	0.003
Peak expiratory flow at spontaneous breathing^a^ (L/min)	5 (5, 7)	8 (5, 10)	−14.15	< 0.001
Peak expiratory flow at extubation^a^ (L/min)	6.5 (6, 8)	21 (9, 32)	−17.99	< 0.001
BIS^c^	69 ± 3	99 ± 1	72.13	< 0.001
SpO_2_^c^	99 ± 1	99 ± 1	0.6909	0.4913
Time to extubation (min)	12 ± 4	6 ± 3	8.993	0.0391
Duration of PACU stay^c^ (min)	49 ± 4	40 ± 5	9.367	< 0.001
Incidence of postoperative cough#	6 (51)	9 (50)	0.776	0.378
Incidence of postoperative pharyngeal pain#	11 (51)	13 (50)	0.274	0.601

#Pearson chi-square test. *Fisher’s exact test. ^a^Mann-Whitney *U*-test. ^b^Wilcoxon rank-sum test. ^c^Independent samples *t*-test

### Secondary outcomes

Then, we assessed the degree of coughing and tracheal tube tolerance, the peak expiratory flow and BIS value at extubation, the SpO_2_ at 2 min after extubation, time to extubation, the duration of PACU stay and laryngeal discomfort complications after extubation. We found that the coughing scores were significantly lower in Group S (1(1, 1)) than in Group C (1(1, 2)), *p* < 0.01 (Table 2). Importantly, the tracheal tube tolerance scores were also significantly better in Group S (0(1, 1)) than in Group C (1(1, 3)), *p* < 0.001 (Table 2). To further verify the reliability of endotracheal tube tolerance after patient sedation, we assessed the incidences of agitation and active extubation. The incidences of agitation and active extubation were significantly lower in Group S (0(51)) than in Group C (8(50) and 5(50), respectively), *p* < 0.05 and *p* < 0.01, respectively (Table 2). We found that the peak expiratory flow at spontaneous breathing and extubation was significantly lower in Group S (5(5, 7) and 6.5(6, 8), respectively) than in Group C (8(5, 10) and 21(9, 32), respectively), *p* < 0.001 (Table 2). At the same time, we assessed the BIS value of the depth of sedation at the time of extubation, the SpO_2_ at 2 min after extubation and the duration of the PACU stay. We found that the BIS value in Group S was lower than that in Group C (*p* < 0.001, Table 2), but the SpO_2_ was not significantly different between the two groups. However, the time to extubation and the duration of PACU stay in group S (12 ± 4 and 49 ± 4, respectively) were longer than those in group C (6 ± 3 and 40 ± 5, respectively), *p* < 0.05 and *p* < 0.001, respectively (Table 2). In addition, we assessed the incidences of postoperative cough and pharyngeal pain, and the results showed that there were no significant differences between Group S and Group C (Table 2).

## Discussion

Coughing in patients with lung disease produces large amounts of aerosols that contain viruses and pathogenic microorganisms, such as *Mycobacterium tuberculosis* [18, 19], *Pseudomonas aeruginosa* [20] and especially SARS-CoV-2 [21]. These aerosols containing viruses and pathogenic microorganisms may spread respiratory diseases, especially COVID-19 [21, 22]. Coughing and peak expiratory flow are high-risk factors for COVID-19 transmission [4, 5]. In the current study, the incidence of induced coughing and in the peak expiratory flow at extubation by BIS-guided sedation was significantly lower than in those with no sedation, which suggests significantly lower aerosol and droplet generation during endotracheal tube extubation in patients in the PACU. Therefore, endotracheal tube removal under BIS sedation might be beneficial for controlling and preventing the transmission of COVID-19.

During recovery from general anaesthesia, sputum aspiration and tracheal tube extraction are the strongest stimulators of the tracheal mucosa and are most likely to induce cough. Although the incidence of coughing reactions was low in Group C after application of compound lidocaine cream combined with tetracaine, 22% of patients still suffered from coughing. A study showed that topical anaesthesia with 0.75% ropivacaine before intubation can significantly reduce the incidence of cough during periextubation, and the incidence of cough can still reach 34.62% [23]. Another study showed that the incidence of cough induced by extubation in patients who received topical anaesthesia with 2% lidocaine before intubation still reached 26.3% [24]. These results were similar to our findings. However, there is still a need to reduce the incidence of extubation-induced cough during the epidemic. Endotracheal tube removal under sedation was first used in the ICU and for special patients’ recovery from anaesthesia. The incidence of choking reaction was significantly reduced by intramuscular dexmedetomidine [8], continuous postoperative infusion of remifentanil at 0.3 μg/kg/min [9] and intravenous infusion of propofol combined with remifentanil [25]. Therefore, it is feasible to remove the endotracheal tube under sedation. Our study showed that the incidence of choking reaction was significantly reduced by BIS-guided sedation, and the incidence was reduced from 11 (50) to 1 (51).

A study showed that the amount of aerosol produced was related not only to cough [1] but also to peak expiratory flow [5] during recovery from general anaesthesia. We further assessed the peak expiratory flow at spontaneous breathing and extubation, and the results suggested that the peak expiratory flow at spontaneous breathing and extubation was significantly lower in the sedation group than in the control group. We speculated that sedation increased patient tolerance to the endotracheal tube, and the cerebral cortex was less responsive to endotracheal tube-induced airway stimulation; therefore, the expiratory flow rate decreased. Almeida C. M. et al. suggested that cough induced by intense airway water stimulation increased peak expiratory flow by 15 L/min compared with spontaneous cough [26]. Ultrafine particles (0.02–1 μm) were generated during peak flow measurement [5], and particles smaller than 5–10 μm have been defined as “aerosols” or “droplet nuclei” and can remain airborne for extended periods of time, travelling greater distances, and can cause transmission by settling into the lower respiratory tract [27]. Furthermore, considerable levels of SARS-CoV-2 RNA were detected in two definite size ranges: submicron particles (0.25–1.0 μm) with concentrations of 9 and 40 copies/m^3^ and supermicron particles (> 2.5 μm) with concentrations of 7 and 9 copies/m^3^, whereas particles ≤ 2.5 μm (fine particles) and ≤ 0.1 μm (ultrafine particles) can reach the lung tissues and settle in the alveolar ducts and sacs [22]. Droplet transmission is commonly reported to occur in particles with diameters > 5 μm that can quickly settle gravitationally on surfaces (1–2 m). Instead, fine and ultrafine particles (airborne transmission) can stay suspended for an extended period of time (≥ 2 h) and be transported further, up to 8 m, through simple diffusion and convection mechanisms [22, 28]. Our study showed that the median peak expiratory flow was reduced from 8 to 5 (L/min) at spontaneous breathing and from 21 to 6.5 (L/min) at extubation, which might be beneficial for preventing the transmission of COVID-19 to medical staff.

During recovery from general anaesthesia, the patient exhibits emergence agitation. The main factor is delayed extubation, and the risk is 16.7 times higher than that of removing the endotracheal tube [29]. L. I. Jing et al. suggested that sedation with dexmedetomidine is as effective as sedation with propofol without affecting the awakening and extubation time of patients [30]. Leonard U. Edokpolo et al. suggested that maintaining sedation at a BIS of 60~70 by combining low-dose dexmedetomidine with propofol can maintain spontaneous breathing [13]. Therefore, we administered an intraoperative pump injection of dexmedetomidine combined with target-controlled infusion of propofol in the PACU for sedation. Our results showed that BIS-guide sedation significantly increased tracheal tube tolerance, and the tracheal tube tolerance scores were reduced to 0 (0, 1) compared to the no sedation group (1(1, 3)). We also found that BIS-guide sedation significantly reduced the incidence of emergence agitation by 16% when compared to the no sedation group. The most serious effect is the voluntary removal of the endotracheal tube during recovery from general anaesthesia in patients with endotracheal intubation. Our study found that BIS-guide sedation significantly reduced the active extubation rate by 10% when compared to the no sedation group. A study showed that remifentanil 0.025–0.05 μg.kg(−1).min(−1) achieves satisfactory tracheal tube tolerance in awake and spontaneously breathing patients when performed under general anaesthesia, and the patients had a score of 3 for the respiratory response indicator of the comfort scale of patients [14]. Another study showed that both dexmedetomidine and remifentanil are effective sedatives for awake intubation, but remifentanil exhibited better tracheal tube tolerance (well tolerated in dexmedetomidine 26% vs. remifentanil 65%) and a moderately increased risk of desaturation [31]. Dexmedetomidine was associated with a longer time to extubation and fewer complications following extubation than tramadol [32]. Aouad M. T. et al. demonstrated that dexmedetomidine (1 μg/kg with total dose 100 μg, 0.5 μg/kg with total dose 50 μg, 0.25 μg/kg with total dose 25 μg) provided the best quality of emergence from general anaesthesia, including the control of cough, agitation, hypertension, tachycardia and shivering at the end of surgery, and the 3 doses did not delay extubation (the average time to extubation: 16~19 min) [33]. Aouad M. T. et al. [33] suggested that the average duration of PACU stay ranged from 58 to 63 min in 3 groups, which was consistent with our results in the sedation group (49 ± 4 min). We found that the duration of PACU stay in the sedation group increased by approximately 23% when compared with the control group (40 ± 5 min). Although the average duration of PACU stay increased by approximately 9 min, this increase was not very significant for routine clinical work.

To resolve whether the combination of two sedatives increases the risk of sedation, the Edokpolo L. study suggested that propofol (1 mg/kg) and a bolus dose of dexmedetomidine (0.3 μg/kg) did not affect the patient’s spontaneous breathing [13]. The total dose of dexmedetomidine in our study was 0.2 μg/kg, which was smaller than the 0.3 μg/kg in Edokpolo L. U. et al.’s study. The dose of propofol to be used (propofol: 0.5~1.5 μg/ml) in our study was 0.817 ± 0.036 mg/kg, which was also no more than Edokpolo L. et al. [13]. Therefore, we observed no side effects of our sedation regimen, and a larger sample size may be needed to further investigate the side effects of our sedation regimen. The administration of dexmedetomidine reduced the amount of propofol used in our study. Whether PACU patients benefit from the sedative effect of dexmedetomidine remains debated. One study confirmed that dexmedetomidine achieved adequate sedation; the ED50 was 0.29 μg/kg, and the ED95 was 0.86 μg/kg [34]. In the literature, the intended level of sedation using dexmedetomidine has been reported to be achieved at doses of 0.2–0.7 μg/kg/h [35]. The elimination half-life (t1/2β) of dexmedetomidine at 1 μg/kg administered over 10 min was 158.16 ± 52.90 min [36]. In our study, dexmedetomidine was infused at 0.2–0.7 μg/kg/h for 30 min. The time from dexmedetomidine infusion to PACU entry was no more than 101 min, which was lower than the elimination half-life (t1/2β) suggested by Xu B. et al. Therefore, it is possible that the sedative effect of dexmedetomidine infusion in this study still exists in PACU, but there is no direct evidence from our study. This is very controversial and needs to be further proved by follow-up studies.

In addition, we assessed the incidences of postoperative cough and postoperative pharyngeal pain, but there was no significant difference between the two groups.

There are 4 limitations to our study. First, this study did not consider the patient’s anxiety state. During recovery from general anaesthesia, the patient exhibits emergence agitation due to poor tolerance to the tracheal tube, which could not be well revealed in this study based on other factors, such as preoperative anxiety or bladder irritation caused by urine retention. Second, this study did not assess the patient’s pain state before extubation because the degree of pain is an important factor for restlessness during recovery. Third, because the infusion of remifentanil was stopped at the end of surgery and remifentanil is a very rapidly metabolized drug, although this study did not assess the total remifentanil consumption, the total remifentanil consumption had almost no effect on the result. Fourth, this study did not reveal that in the setting of extubation of the trachea, neither the distribution of exhaled gases nor the capacity of these gases to carry virus in the peri-extubation period has been fully quantified. The reduction in peak expiratory flow at spontaneous breathing and at extubation reduces the number of aerosols produced and the flight distance to prevent the spread of respiratory infectious diseases, which is unreliable, and further research is needed in the future.

## Conclusion

Our study demonstrated that the application of BIS-guided sedation with dexmedetomidine and propofol inhibited the coughing reaction caused by sputum suction or tracheal tube removal during recovery from general anaesthesia, reduced peak expiratory flow at spontaneous breathing and at extubation and increased the body’s tolerance to the tracheal tube. Therefore, BIS-guided sedation with dexmedetomidine and propofol may play an important role in preventing medical staff from contracting respiratory infectious diseases, especially during the COVID-19 epidemic.

1. *What is known*

- BIS-guided sedation with dexmedetomidine and propofol exerted a better effect in preventing cough reactions caused by tracheal extubation.
- BIS-guided sedation with dexmedetomidine and propofol exerted a better effect in reducing peak expiratory flow during recovery from general anaesthesia.

2. *What is new*

- BIS-guided sedation with dexmedetomidine and propofol may play an important role in preventing medical staff from contracting COVID-19 in the PACU.
- BIS-guided sedation was performed by infusing dexmedetomidine in the operating room for 30 min combined with an infusion of propofol at 0.5~1.5 μg/ml to maintain a BIS value of 60–70 in the PACU, which was a safe method to achieve sedation and maintain spontaneous breathing.

## Data Availability

The datasets generated and/or analysed during the current study are not publicly available due to limitations of ethical approval involving the patient data and anonymity but are available from the corresponding author on reasonable request (the corresponding author is Prof. *Erfei Zhang*, Department of Anesthesiology, the Affiliated Hospital of Yan’an University, Yan’an 716000, Shaanxi Province, P. R. China, Tel: + 86 0911 2881264, e-mail: zhangerfei09@126.com).
